# Deep learning-based quantitative histopathology of endoscopic biopsies in Crohn’s disease: a retrospective cross-sectional validation study

**DOI:** 10.3389/fimmu.2026.1841261

**Published:** 2026-06-05

**Authors:** Xin Jiang, Pan Li, Zhaojing Chen, Hui Yao, Hao Jia, Taiping Wang, Xuefeng Tang

**Affiliations:** 1 Department of Pathology, Chongqing Academy of Medical Sciences, Chongqing General Hospital, Chongqing University, Chongqing, China; 2 Department of Anesthesiology, Children’s Hospital of Chongqing Medical University, National Clinical Research Center for Children and Adolescents’ Health and Diseases, Ministry of Education Key Laboratory of Child Development and Disorders, Chongqing Key Laboratory of Child Neurodevelopment and Cognitive Disorders, Chongqing, China

**Keywords:** artificial intelligence, Crohn’s disease, deep learning, endoscopic biopsy, quantitative histopathology

## Abstract

**Background:**

Crohn’s disease (CD) shows marked histopathologic heterogeneity, and conventional histologic assessment remains dependent on subjective visual evaluation, limiting reproducibility and objective quantification. We aimed to develop and clinically validate a deep learning-based framework for quantitative assessment of endoscopic biopsies in CD.

**Methods:**

We retrospectively analyzed 3,641 endoscopic biopsy slides from 687 patients with CD using a hierarchical deep learning workflow implemented on the Palgo platform. This workflow integrated tissue-compartment segmentation, crypt-level segmentation and classification, and inflammatory cell detection and classification. From the resulting whole-slide outputs, we derived quantitative histologic metrics, including cryptitis and crypt-abscess ratios, mucosal and submucosal inflammatory cell densities, hotspot-based density measures, and mucosal stromal proportion. Concordance with the pathologist assessment was evaluated using concordance correlation coefficients, and associations with clinical indicators were examined using Spearman’s rank correlation coefficients.

**Results:**

The models showed robust internal performance across the main segmentation and classification tasks, with Dice coefficients and AUC values generally exceeding 0.95. AI-derived crypt abscess and cryptitis ratios showed good concordance with pathologist assessment, whereas agreement was weaker for certain cell-level outputs, particularly submucosal plasma cell density (CCC = 0.376). Grade-stratified calibration indicated a tendency toward AI over-detection in submucosal regions with negative or low manual scores, underscoring the need for cautious interpretation of this metric. Several AI-derived features were associated with clinical or endoscopic activity; however, these associations were generally weak to moderate and should be regarded as exploratory. In particular, crypt abscess ratio was associated with endoscopic activity; submucosal eosinophil density and the mucosa-to-submucosa plasma cell ratio were associated with CDAI; and mucosal stromal proportion was associated with CRP. Exploratory group comparisons further suggested greater submucosal inflammatory infiltration in CD biopsies than in the heterogeneous non-CD inflammatory colitis group.

**Conclusions:**

This AI-based framework provides objective and reproducible tissue-level quantification of CD histopathology and may complement routine pathologic assessment. By integrating structured quantitative outputs with heatmap-based visual review, it offers a practical pathologist-supervised approach for standardizing histologic evaluation in CD.

## 1 Introduction

Crohn’s disease (CD), a chronic transmural form of inflammatory bowel disease (IBD), has a rising global incidence and imposes a substantial burden of long-term morbidity and complications (1). Accurate diagnosis and disease characterization are essential for optimizing therapy, informing surgical decision-making, and improving prognosis. In this diagnostic workflow, histopathologic examination of endoscopic biopsy specimens is pivotal, offering critical evidence to differentiate Crohn’s disease from ulcerative colitis, infectious enteritis, and other inflammatory mimics (2, 3). In contrast to UC, where inflammation is usually continuous and diffusely distributed within the mucosa, CD is often marked by patchy, focal, and spatially heterogeneous inflammation that may extend across different tissue compartments (4–6). This heterogeneity limits the reliability of conventional assessment based on small endoscopic biopsy samples and provides a rationale for CD-specific computational pathology approaches (7, 8).

Conventional histopathologic assessment faces challenges due to the complex, heterogeneous, and dynamic microscopic changes that characterize Crohn’s disease (3, 9). Current practice is predominantly dependent on the visual interpretation of individual pathologists, resulting in significant inter-observer variability, especially in identifying subtle but clinically significant features such as focal chronic inflammation, crypt architectural distortion, basal plasmacytosis, and granulomas in limited biopsy samples (10). This issue is especially relevant in CD, where diagnostically important abnormalities may be patchy and unevenly distributed within the mucosa and, when sampled, the submucosal compartment (8). As a result, conventional semi-quantitative grading may not adequately reflect the spatial distribution of inflammation, the severity of involvement in different tissue compartments, or the cellular composition of microscopic inflammatory infiltrates. This subjectivity increases diagnostic uncertainty and obstructs reproducible, standardized measurement of microscopic disease activity. Parallel efforts to standardize endoscopic scoring conventions in CD further highlight the need for harmonized and responsive activity measures across modalities (9, 11).

The emergence of computational pathology, driven by foundational AI models such as UNI and CONCH and more recent pathology foundation models (12–16), has initiated a paradigm shift in digital histopathology. Deep convolutional neural networks, originally designed for image classification tasks such as breast cancer detection, are increasingly being adapted for gastrointestinal and IBD pathology (11, 16). These technologies facilitate rapid slide-level analysis and can underpin large-scale, standardized evaluation of IBD biopsies (11, 13, 16, 17). Recent studies in IBD have demonstrated that deep learning models can accurately assess histologic disease activity, produce quantitative histologic scores, and support qualitative reporting in both ulcerative colitis and Crohn’s disease (18–23). However, most AI-based pathology studies in IBD have focused on UC or on overall mucosal activity grading, where inflammatory changes are typically more continuous and diffusely distributed. By comparison, studies applying AI specifically to CD have more often addressed diagnostic or prognostic tasks, such as differentiating CD from intestinal tuberculosis or predicting postoperative outcomes, rather than interpretable quantification of CD-related microscopic features at the compartment-specific level (20, 21, 23, 24).

This represents an important methodological and clinical gap. In CD, histopathologic assessment often requires more than a diagnostic label or a global activity score. It also depends on determining the anatomic distribution of inflammation, the degree of involvement within available tissue compartments, the presence of crypt injury, and the spatial pattern of inflammatory cell populations. These features are difficult to measure reproducibly by routine visual assessment, particularly in limited biopsy specimens where diagnostically relevant abnormalities may be focal or unevenly distributed. Therefore, an interpretable AI framework combining tissue-compartment segmentation, crypt-level lesion analysis, inflammatory cell classification, and spatial density estimation may provide quantitative information that complements conventional pathological assessment and supports more standardized evaluation of microscopic disease activity in CD (2, 9, 18–22).

To address this gap, we developed an interpretable AI framework for fine-grained quantitative analysis of CD endoscopic biopsies. Rather than limiting the model to global classification, we designed a hierarchical deep learning-based pipeline to localize available tissue compartments, including the mucosa and, when present, the submucosa, and to identify distinct pathologic features such as crypt abscesses and inflammatory infiltrates. Building on these outputs, we engineered a set of objective quantitative metrics-such as compartment-specific inflammatory cell densities and mucosal stromal proportions-to complement and refine conventional semi-quantitative grading. Finally, we explored the clinical relevance of these AI-derived metrics by examining their concordance with pathologist assessments and their correlations with established disease activity indicators, including the Crohn’s Disease Activity Index (CDAI) and C-reactive protein (CRP). By incorporating multi-scale, compartment-specific, and cell-level histopathologic features into a unified analytical framework, this study aims to provide a quantitative pathology layer that may complement routine assessment and support more objective evaluation of CD activity.

## 2 Methods

### 2.1 Study design and data sources

This retrospective, single-center investigation examined patients diagnosed with Crohn’s disease (CD) between January 2021 and December 2024. Consecutive hematoxylin-eosin-stained endoscopic biopsy specimens from patients with a confirmed diagnosis of Crohn’s disease were retrieved from the pathology archive and digitized as whole-slide images (WSIs) at a spatial resolution of 0.23 µm per pixel. All slides were processed in the same pathology department according to routine institutional protocols and scanned using a standardized digital scanning workflow.

Clinical data, including demographic characteristics, simplified Crohn’s Disease Activity Index (CDAI) scores, C-reactive protein (CRP) values, endoscopic activity, and treatment information, were extracted from the electronic medical record. Each slide was independently reviewed by two gastrointestinal pathologists with expertise in inflammatory bowel disease. Discrepancies in histologic assessment were resolved by consensus. These expert assessments provided the clinicopathologic reference diagnosis and manual evaluation framework for comparison with AI-derived quantitative metrics. Task-specific annotations used for model training and validation are described below.

### 2.2 Study population and slide selection

The initial cohort consisted of 687 patients with CD and 3, 641 endoscopic biopsy slides. Inclusion criteria were: (1) histologically and clinically confirmed CD; (2) available endoscopic, imaging, and follow-up data consistent with the diagnosis; (3) biopsies from at least five anatomic sites, including the terminal ileum and rectum; and (4) sufficient evaluable tissue for histopathologic assessment, with mucosal tissue required and submucosal tissue analyzed when identifiable and evaluable.

Exclusion criteria were: (1) lack of diagnostic consensus between the two pathologists and (2) major technical artifacts that impaired histologic assessment, such as excessive section thickness (>5 µm), prominent knife marks, crush artifacts, severe tissue folding, out-of-focus regions, or poor staining quality.

After quality control, 687 patients and 3,641 slides met eligibility criteria. For model development and validation, patients were randomly allocated to training and validation sets in a 4:1 ratio using stratified sampling to preserve the distribution of histologic severity. The split was performed at the patient level before region-of-interest or patch extraction. Therefore, all WSIs, annotated regions, and image patches derived from the same patient were retained within the same dataset subset, preventing patient-level leakage between training and validation data. After model selection, the trained models were applied to eligible WSIs to generate AI-derived quantitative histologic metrics. These metrics were then linked with available clinical variables, including CDAI, CRP, endoscopic activity, and routine laboratory data, for clinicopathologic correlation analyses.

For exploratory comparison, we also included a non-Crohn’s inflammatory colitis group. This group consisted of patients with inflammatory colitis other than CD, including ulcerative colitis, infectious colitis, ischemic colitis, drug-induced colitis, nonspecific chronic colitis, and other inflammatory conditions, as determined by the final clinicopathologic diagnosis. The non-Crohn’s inflammatory colitis cases were not used for model development and were analyzed only after model selection to explore compartment-specific inflammatory patterns. This analysis was not designed to develop or validate a diagnostic classifier.

### 2.3 AI workflow and model architecture

**Figure 1:**
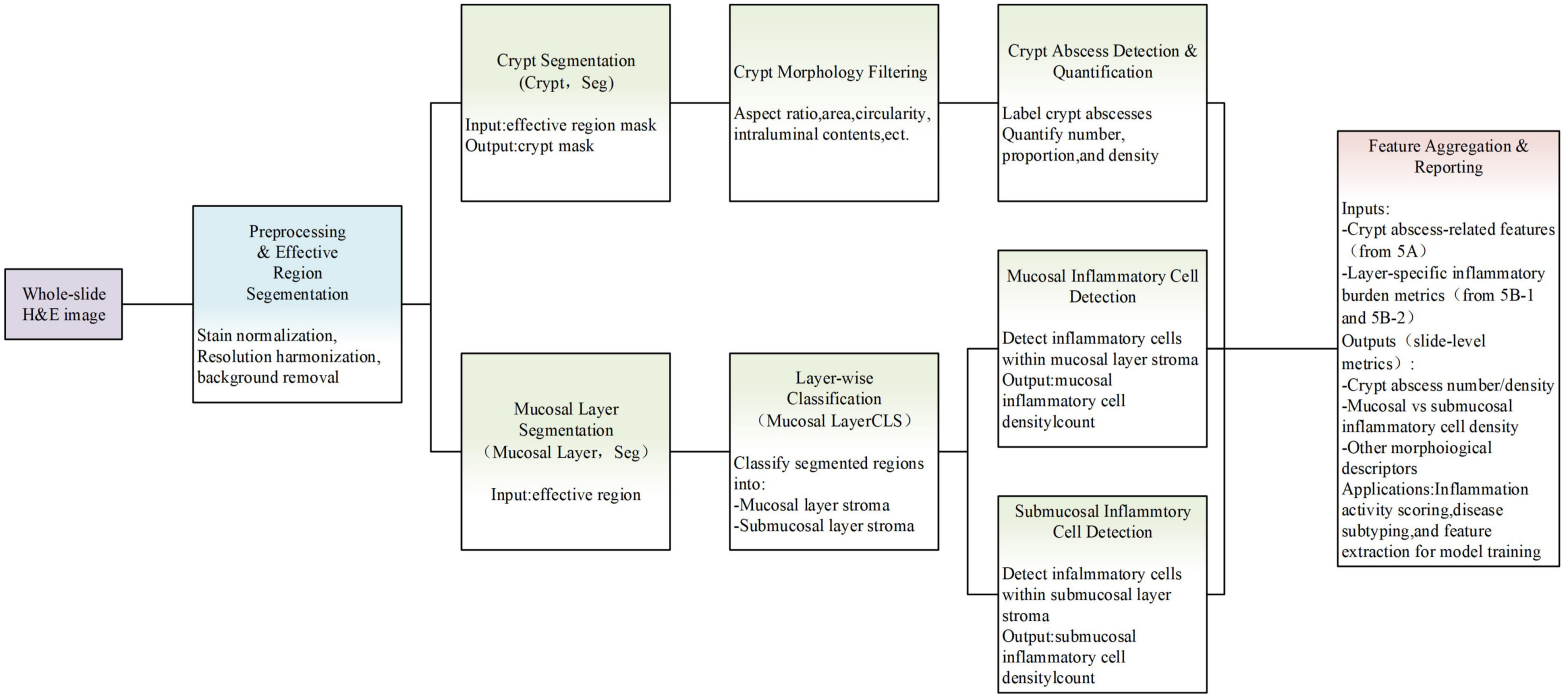
Workflow of the Palgo-based histopathology analysis platform. WSI, whole-slide image; Seg, segmentation; CLS, classification; ML, mucosal layer; SL, submucosal layer. The diagram illustrates the module-level workflow of the Palgo-based platform for gastrointestinal biopsy WSI analysis. Effective region segmentation generates the tissue mask for downstream crypt and mucosal layer segmentation. These outputs are then used for stromal region derivation and crypt morphology filtering. Solid blue lines denote sequential inputs, blue dotted lines denote shared inputs, and green dashed lines denote stromal region derivation. Terminal nodes indicate the final outputs for quantitative analysis: crypt abscesses, mucosal inflammatory cells, and submucosal inflammatory cells.

Whole-slide images were analyzed using the Palgo platform, a digital pathology AI framework for annotation management, model training, inference, and quantitative feature extraction (22, 25, 26). In the present study, Palgo served as the implementation environment for task-specific models rather than as an unspecified black-box scoring tool. The workflow included three hierarchical levels: low-magnification tissue-compartment segmentation, intermediate-magnification crypt analysis, and high-magnification inflammatory cell detection and classification (**Figure 1**).

The analytical workflow was implemented across tissue, crypt, and cellular scales. For tissue-compartment segmentation, an EfficientNet-B2–UNet model delineated the mucosa, submucosa, and muscularis mucosae, generating an anatomical framework for subsequent region-specific analyses (27). Crypt structures were then identified and categorized as normal crypts, cryptitis, or crypt abscesses using a dual-branch network. In this model, one branch assigned crypt-level classifications, whereas the other assessed neutrophil infiltration within crypt lumens, thereby improving the discrimination of cryptitis and crypt abscesses (11). For cell-level assessment, inflammatory cells in the mucosal and submucosal compartments, including plasma cells, lymphocytes, neutrophils, and eosinophils, were detected and classified using an EfficientNet-B1-UNet-based model (26, 28).

### 2.4 Annotation, model training, and technical validation

A structured three-stage process was employed to conduct manual annotation, which involved initial labeling, expert review, and quality control based on archived pathology records. Pathologists annotated representative regions of the mucosa, submucosa, muscularis mucosae, and background on whole-slide images for tissue segmentation. In order to conduct crypt-level analysis, representative regions that contained crypt structures were identified and classified as normal crypts, cryptitis, or crypt abscesses in accordance with predetermined histologic criteria.

Rather than conducting an exhaustive manual annotation of each cell throughout the entire WSI, the reference standard for inflammatory cell detection at high magnification was established using representative image regions. Mucosal and submucosal regions exhibiting varying degrees of inflammatory activity were sampled using high-power patches. In these regions, gastrointestinal pathologists identified inflammatory cells, such as plasma cells, lymphocytes, neutrophils, and eosinophils. The final labels were determined by consensus, and cells with equivocal morphology or discrepant labels were further reviewed. The inflammatory cell detection model was trained and validated using these patch-based annotations as the ground truth. For each analytical task, cases were divided into training and validation sets at the patient level before extracting regions of interest or image patches. Accordingly, all WSIs, annotated areas, and derived patches from a given patient were assigned to the same dataset subset. This strategy was adopted to avoid patient-level data leakage between the training and validation cohorts.

Model development began with self-supervised pretraining on a large collection of unlabeled hematoxylin-eosin-stained pathology images. The pretrained weights were then fine-tuned using task-specific annotated images from the present cohort. During local model development, all model weights remained trainable rather than being frozen. The models that achieved the best performance on the internal validation sets were subsequently used for slide-level inference and extraction of quantitative metrics.

All WSIs were prepared from hematoxylin-eosin-stained sections processed in the same pathology department and scanned under a standardized protocol at a resolution of 0.23 µm per pixel. Although no formal stain normalization procedure was applied, slide-level quality control was performed to exclude slides with poor staining quality, severe tissue folding, prominent knife marks, crush artifacts, or out-of-focus areas. Color augmentation was incorporated during model training to improve robustness to variability in H&E staining. Given the single-center design of this study, further external validation using slides from different institutions and scanner platforms is needed to evaluate the generalizability of the models.

After training, the inflammatory cell detection model was applied to WSIs in a tile-based manner within the previously segmented mucosal and submucosal compartments. The coordinates and predicted classes of detected cells were then aggregated to calculate inflammatory cell densities at both whole-compartment and hotspot levels. Thus, the WSI-level cell-density metrics represented quantitative outputs generated by slide-wide model inference, rather than manual extrapolation from selected image patches.

### 2.5 Quantitative histologic metrics

Quantitative histologic metrics were derived from the model outputs to capture major histopathologic features of Crohn’s disease (7, 22). The resulting measurements included the crypt abscess ratio, cryptitis ratio, mucosal inflammatory cell density, submucosal inflammatory cell density, and mucosal stromal ratio.

The crypt abscess ratio was calculated as the number of crypts containing abscesses divided by the total number of evaluable crypts. The cryptitis ratio represented the proportion of evaluable crypts showing cryptitis, with crypt abscesses included in this category. Mucosal inflammatory cell density was determined by quantifying plasma cells, lymphocytes, neutrophils, and eosinophils within the mucosal compartment. This density was reported both across the entire mucosal area and within predefined 500-μm high-density hotspot regions. The same approach was used for the submucosal compartment, where inflammatory cell density was summarized as whole-compartment averages and hotspot values. The mucosal stromal ratio was calculated as the proportion of stromal area within the mucosa and was used to reflect structural remodeling, fibrosis, and crypt atrophy. Submucosal inflammatory cell density was calculated only for slides with an identifiable and evaluable submucosal compartment. When the submucosa was absent or not evaluable, submucosa-based metrics were recorded as not assessable rather than imputed as zero.

To account for sparse and spatially heterogeneous inflammatory cell distributions, kernel density estimation implemented in scikit-learn was applied to the detected inflammatory cell coordinates. This procedure generated smoothed density maps for subsequent quantitative analysis. All histologic metrics were automatically extracted from WSIs, with no manual *post hoc* adjustment.

### 2.6 Statistical analysis

Agreement between AI-derived measures and pathologist assessments was evaluated using concordance correlation coefficients (CCCs). CCCs were calculated for cryptitis, crypt abscesses, and compartment-specific inflammatory cell scores. Spearman’s rank correlation coefficients were used to analyze associations between AI-derived quantitative histologic metrics and clinical indicators, including simplified Crohn’s Disease Activity Index scores, C-reactive protein levels, and other routine biochemical variables. Analyses involving submucosal metrics were restricted to slides with identifiable submucosa and paired non-missing AI-derived and manual assessments. For submucosal plasma cell density, an exploratory standardized Bland-Altman analysis was performed using the same evaluable paired cases included in the concordance analysis.

In exploratory subgroup analyses, AI-derived metrics were compared between Crohn’s disease and non-Crohn’s inflammatory colitis cases to assess their potential discriminatory value. Two-sided p-values < 0.05 were considered statistically significant. Statistical analyses were performed using R version 4.3.1 and Python packages including scikit-learn and SciPy. This retrospective observational study was reported in accordance with the STROCSS 2025 guidelines (29).

## 3 Results

### 3.1 Patient cohort and overview of the AI pipeline

**Figure 2:**
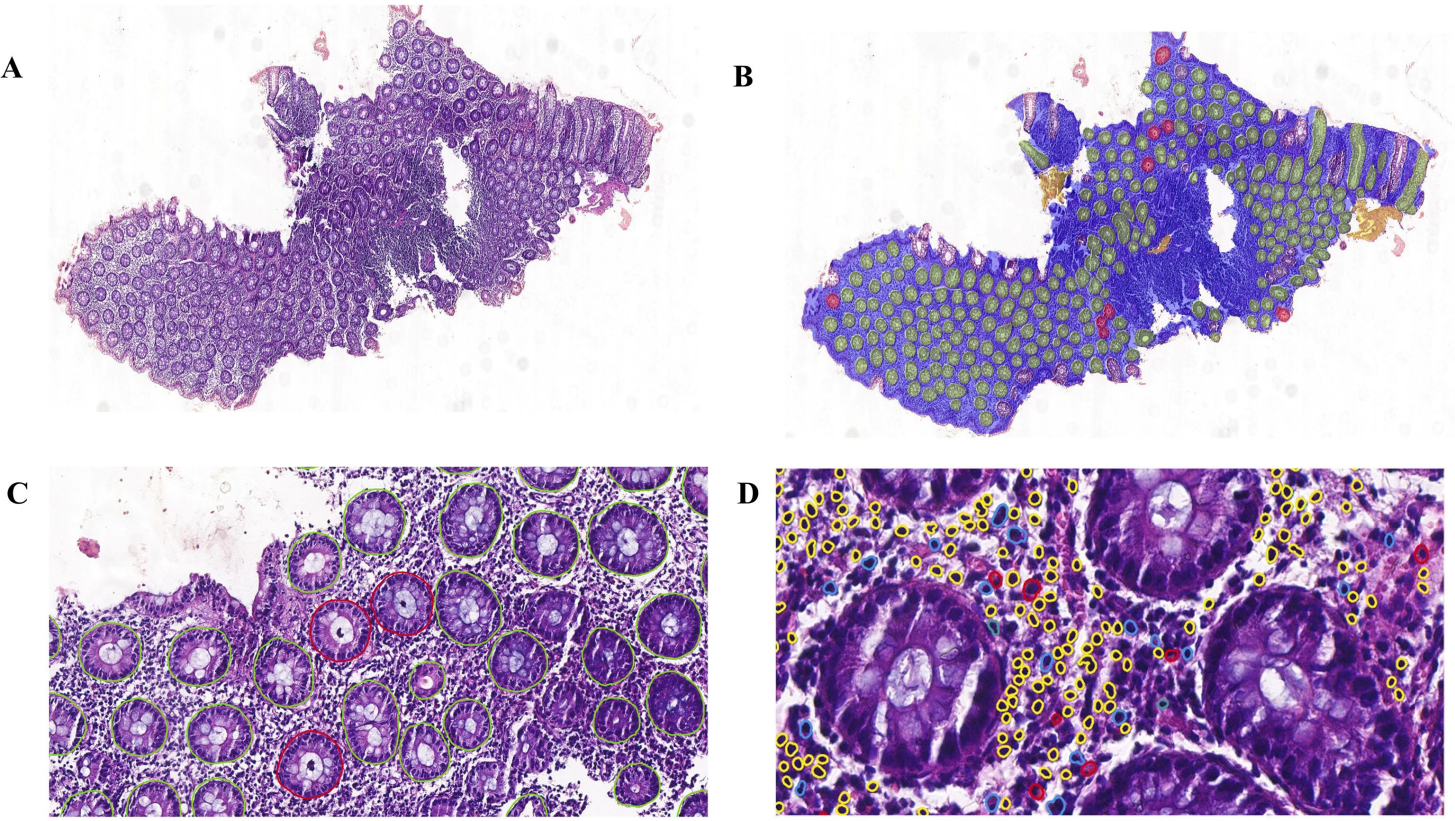
Representative outputs of the segmentation models. **(A)** Original whole-slide image (WSI). **(B)** Low-magnification segmentation of the mucosal layer and muscularis mucosae: yellow, muscularis mucosae; blue, mucosal stroma; green, normal crypts; red, crypt abscesses; purple, cryptitis. **(C)** Medium-magnification view of crypt segmentation: green, normal crypts; red, crypt abscesses. **(D)** High-magnification view of cell-level segmentation: yellow, lymphocytes; blue, plasma cells; red, eosinophils; green, neutrophils. WSI, whole-slide image. The figure illustrates a representative gastrointestinal biopsy analyzed by the AI-based segmentation pipeline. Panel **(A)** shows the original hematoxylin-eosin-stained WSI. Panel **(B)** presents a low-magnification overlay of segmentation results for tissue compartments and crypts, with color-coded masks indicating muscularis mucosae, mucosal stroma, normal crypts, crypt abscesses, and cryptitis. Panel **(C)** demonstrates a medium-magnification view focusing on crypt boundaries and the distinction between normal crypts and crypt abscesses. Panel **(D)** displays a high-magnification view with cell-level detection, where overlaid markers indicate lymphocytes, plasma cells, eosinophils, and neutrophils used for subsequent quantitative analyses.

A total of 687 patients with Crohn’s disease and 3,641 endoscopic biopsy slides were incorporated into the analysis. Baseline clinical characteristics are summarized in **Table 1**. **Figure 2** depicts exemplary outputs of the hierarchical AI workflow. At the entire-slide level, the mucosa and submucosa were precisely delineated (**Figures 2A, B**). At increased magnification, individual crypts were clearly delineated and annotated, and inflammatory cells were identified within and surrounding the crypts (**Figures 2C, D**), forming the foundation for subsequent quantitative analyses.

**Table 1:** Baseline demographic and clinical characteristics of the study cohort (n = 687).

Characteristics	Number of patients, mean ± SD, or n (%)
Total number	687
Age (years)	28.4 ± 8.9
≤16 years	64(9.31%)
17–40 years	566(82.39%)
>40 years	57(8.30%)
Gender	
Male	454(66.09%)
Female	233(33.91%)
Disease duration (years)	3.8 ± 2.6
<5 years	538(78.31%)
≥5 years, <10 years	121(17.61%)
≥10 years	28(4.08%)
Disease location	
Terminal ileum	37(5.39%)
Colon	47(6.84%)
Ileocolonic	548(79.77%)
Ileocolonic + upper gastrointestinal	55(8.01%)
Endoscopic Score (SES-CD)	
0–2 points	96(13.97%)
3–6 points	100(14.56%)
7–15 points	227(33.04%)
≥16 points	264(38.43%)
Simplified Crohn’s Disease Activity Index (CDAI)	
≤4 points	0
5–7 points	93(13.54%)
8–16 points	511(74.38%)
>16 points	83(12.08%)
C-reactive protein (CRP)	
Normal (0-10mmol/L)	130(18.92%)
Elevated(>10mmol/L)	557(81.08%)
Treatment	
No treatment	47(6.84%)
5-Aminosalicylic acid derivatives	354(51.51%)
Immunosuppressants	185(26.93%)
Glucocorticoids	269(39.16%)
Biologics	650(94.61%)

SES-CD, Simple Endoscopic Score for Crohn’s Disease; CDAI, Simplified Crohn’s Disease Activity Index; CRP, C-reactive protein.

Values are presented as mean ± standard deviation (SD) or number (percentage), as appropriate.

### 3.2 Segmentation and classification performance

**Figure 3:**
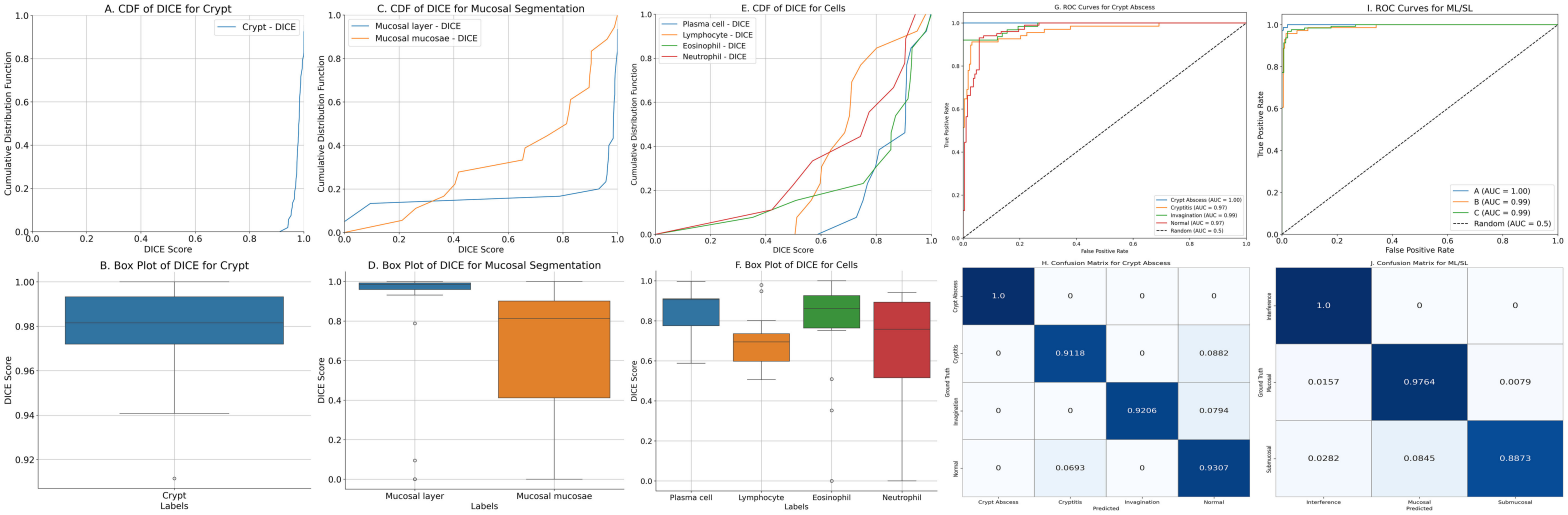
Quantitative performance of core segmentation and classification models. **(A)** Cumulative distribution function (CDF) of Dice similarity coefficient (DICE) values for the crypt segmentation model. **(B)** Box plot of DICE values for the crypt segmentation model. **(C)** CDF of DICE values for the mucosal layer segmentation model. **(D)** Box plot of DICE values for the mucosal layer segmentation model. **(E)** CDF of DICE values for the stromal inflammatory cell segmentation model (mucosal and submucosal layers combined). **(F)** Box plot of DICE values for the stromal inflammatory cell segmentation model. **(G)** Receiver operating characteristic (ROC) curve of the crypt abscess classification model. **(H)** Confusion matrix of the crypt abscess classification model. **(I)** ROC curve of the mucosal layer classification model. **(J)** Confusion matrix of the mucosal layer classification model. CDF, cumulative distribution function; DICE, Dice similarity coefficient; ROC, receiver operating characteristic; ML, mucosal layer; SL, submucosal layer; AUC, area under the curve. Panels **(A–F)** summarize segmentation performance for crypts, mucosal structures, and stromal inflammatory cells, with CDF plots **(A, C, E)** showing the distribution of DICE values across test tiles and box plots **(B, D, F)** depicting median and interquartile ranges. Panels **(G, I)** present ROC curves for the crypt abscess and ML/SL classification models, respectively, and panels **(H, J)** show the corresponding confusion matrices, where rows represent ground truth labels and columns represent model predictions. Curves approaching the upper-left corner, higher AUC values, and DICE scores close to 1.0 indicate better model performance.

**Figure 3**; **Table 2** present the quantitative performance of the segmentation and classification models. For crypt segmentation, the Dice coefficients were close to 1.0, and the interquartile range was small (**Figures 3A, B**). Segmentation of the mucosal and submucosal compartments also yielded high Dice scores, with the majority of values exceeding 0.9 (**Figures 3C, D**). Dice coefficients for inflammatory cell segmentation were marginally reduced; however, they remained adequate for plasma cells, lymphocytes, neutrophils, and eosinophils (**Figures 3E, F**).

**Table 2:** Statistical performance metrics of model results.

Label	Sensitivity	Specificity	FPR	TPR	NPV	PPV	Precision	Recall	ACC	AUC	AP
ML/SL segmentation	0.840	0.962	0.038	0.840	0.527	0.992	0.992	0.840	0.859	–	–
Mucosal mucosae	0.827	0.994	0.006	0.827	0.989	0.909	0.909	0.827	0.984	–	–
Crypt	0.954	0.998	0.002	0.954	0.987	0.994	0.994	0.954	0.988	–	–
Plasma cell	0.855	0.989	0.011	0.855	0.983	0.902	0.902	0.855	0.975	–	–
Lymphocyte	0.653	0.995	0.005	0.653	0.987	0.823	0.823	0.653	0.982	–	–
Eosinophil	0.848	0.999	0.001	0.848	0.999	0.860	0.860	0.848	0.998	–	–
Neutrophil	0.614	0.999	0.001	0.614	0.999	0.812	0.812	0.614	0.998	–	–
Interference	1.000	0.980	0.020	1.000	1.000	0.949	0.949	1.000	0.960	1.000	0.999
Mucosal	0.976	0.959	0.041	0.976	0.979	0.954	0.954	0.976	0.960	0.994	0.993
Submucosal	0.887	0.995	0.005	0.887	0.962	0.984	0.984	0.887	0.960	0.991	0.980
Crypt Abscess	1.000	1.000	0.000	1.000	1.000	1.000	1.000	1.000	0.939	1.000	1.000
Cryptitis	0.912	0.969	0.031	0.912	0.973	0.899	0.899	0.912	0.939	0.966	0.932
Invagination	0.921	1.000	0.000	0.921	0.979	1.000	1.000	0.921	0.939	0.986	0.969
Normal	0.931	0.943	0.057	0.931	0.963	0.895	0.895	0.931	0.939	0.971	0.935

ML, mucosal layer; SL, submucosal layer; FPR, false-positive rate; TPR, true-positive rate; NPV, negative predictive value; PPV, positive predictive value; ACC, accuracy; AUC, area under the curve; AP, average precision.

Sensitivity, specificity, false-positive rate (FPR), true-positive rate (TPR), negative predictive value (NPV), positive predictive value (PPV), precision, recall, accuracy (ACC), area under the receiver operating characteristic curve (AUC), and average precision (AP) are reported for each label. A dash indicates that AUC or AP was not applicable for a given task.

Receiver operating characteristic curves exhibited robust discriminatory ability in classification tasks. The area under the curve (AUC) exceeded 0.95 for crypt abscesses, cryptitis, normal crypts, and other crypt classifications (**Figure 3G**), as well as for mucosal versus submucosal compartments (**Figure 3I**). The confusion matrices demonstrated high recall across all crypt classes, with recall exceeding 90% for the majority of categories and reaching 88.7% for the submucosal compartment (**Figures 3H, J**; **Table 2**).

**Figure 4:**
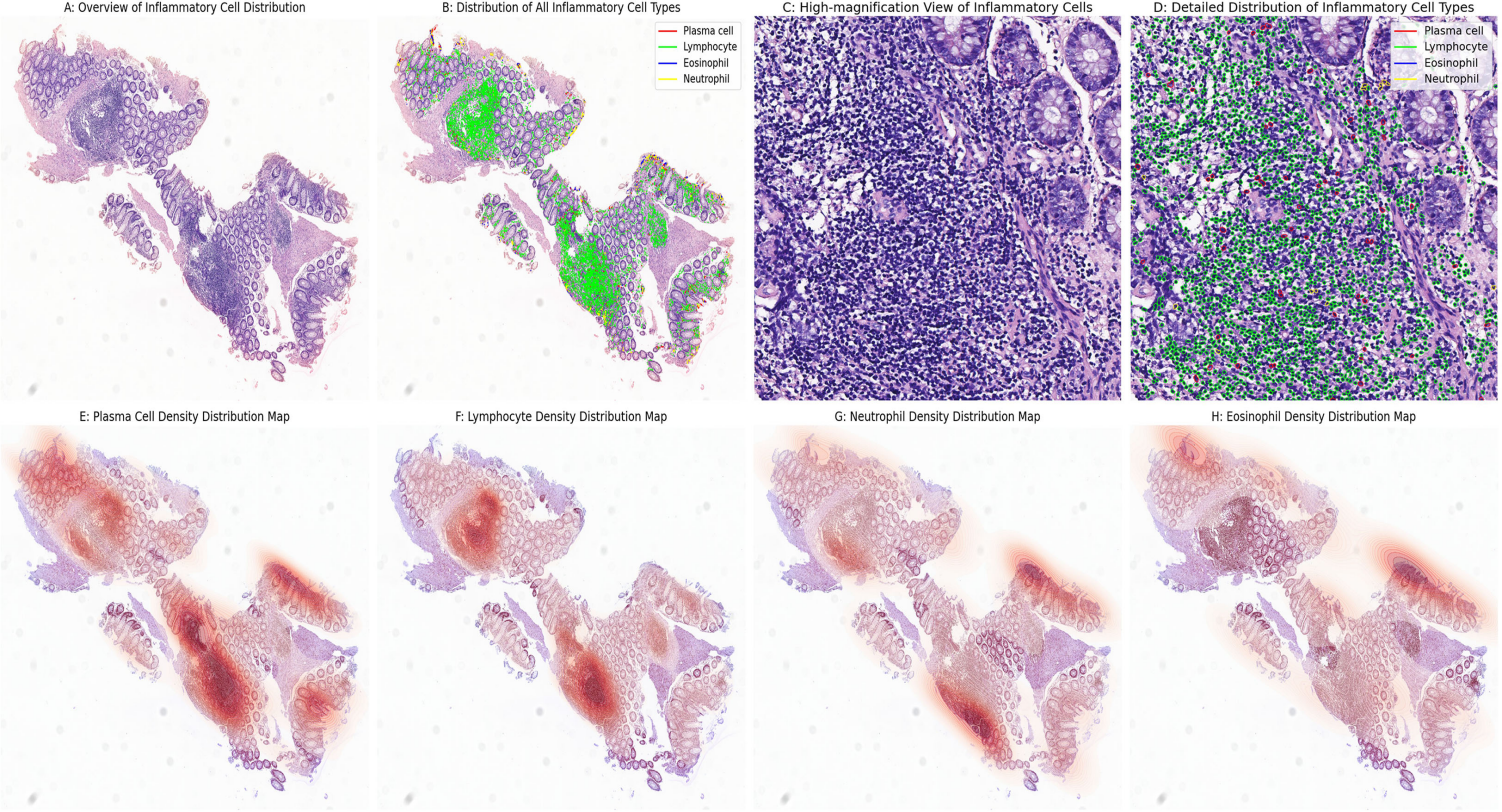
Identification of high-density regions of stromal inflammatory cells. **(A)** Original WSI. **(B)** Full-field segmentation of stromal inflammatory cell localization. **(C)** High-magnification view of the original cellular image. **(D)** High-magnification view of the corresponding cell detection results. **(E)** Heatmap of plasma cell nuclear density, where red denotes higher plasma cell density. **(F)** Heatmap of lymphocyte nuclear density, where red denotes higher lymphocyte density. **(G)** Heatmap of eosinophil nuclear density, where red denotes higher eosinophil density. **(H)** Heatmap of neutrophil nuclear density, where red denotes higher neutrophil density. WSI, whole-slide image. Panels **(A–D)** illustrate the transition from a global view of the biopsy to detailed cell-level detection, while panels **(E–H)** show kernel-based density maps for each inflammatory cell type. Density estimation is restricted to the mucosal and submucosal stromal compartments, and regions with warmer color gradients represent high-density inflammatory foci used for subsequent quantitative analysis.

Kernel density-based maps illustrated the spatial distribution of inflammatory cells (**Figure 4**). Regions of high modeled cell density overlapped with visually inflamed areas on the original hematoxylin-eosin slides, both at low magnification and on zoomed-in views, for plasma cells, lymphocytes, neutrophils, and eosinophils (**Figures 4A–H**).

### 3.3 Agreement between AI-derived metrics and pathologist grading

**Figure 5:**
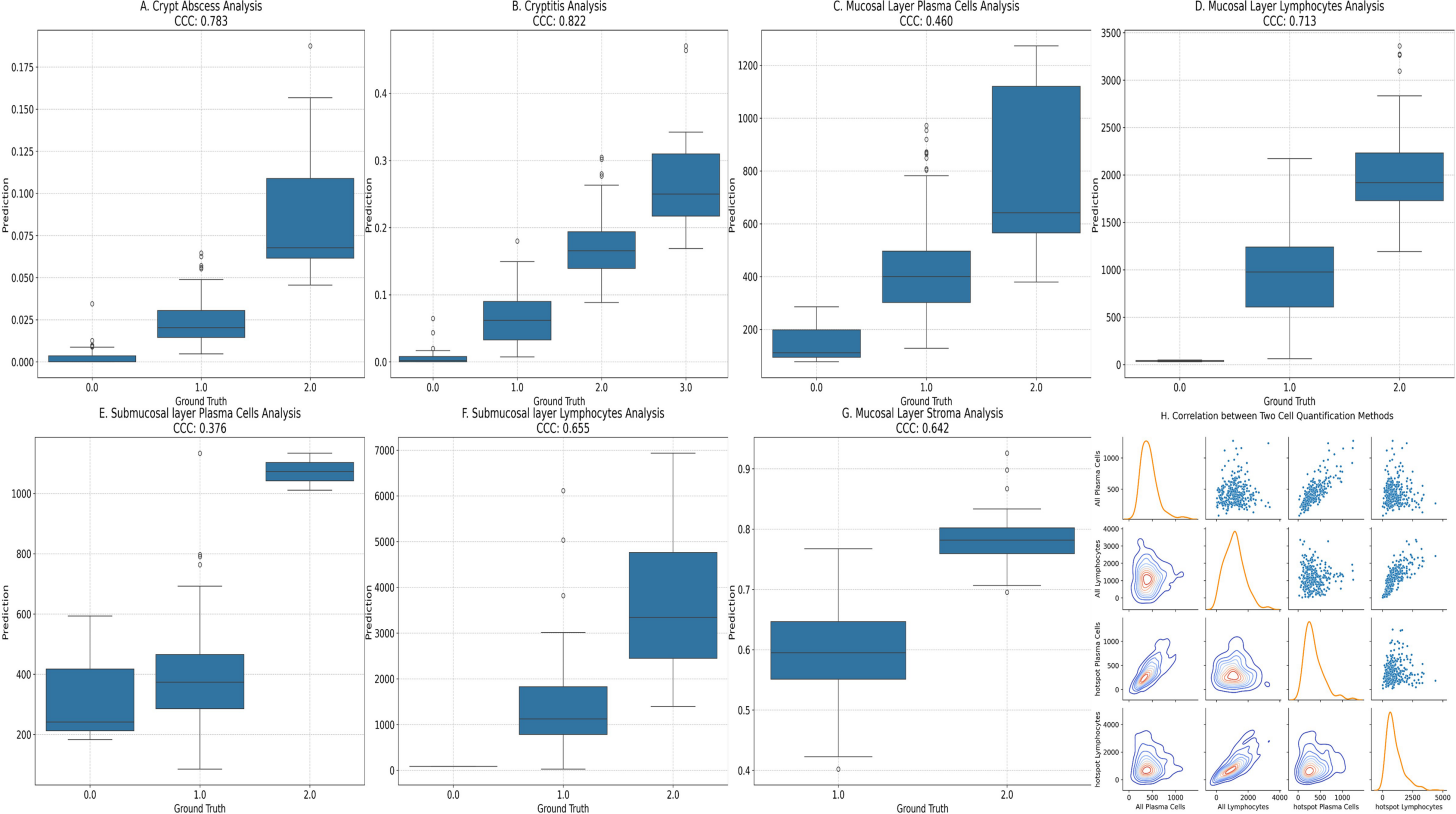
Concordance between AI metrics and manual assessment. **(A)** Concordance of crypt abscess counts between the algorithm and manual assessment (Lin’s concordance correlation coefficient [CCC] = 0.783). **(B)** Concordance of cryptitis counts (CCC = 0.822). **(C)** Concordance of plasma cell counts per unit area in the mucosal layer (CCC = 0.463). **(D)** Concordance of lymphocyte counts per unit area in the mucosal layer (CCC = 0.713). **(E)** Concordance of plasma cell counts per unit area in the submucosal layer (CCC = 0.376). **(F)** Concordance of lymphocyte counts per unit area in the submucosal layer (CCC = 0.655). **(G)** Concordance of mucosal stromal proportion, an index of stromal fibrosis and crypt atrophy (CCC = 0.642). **(H)** Relationship between average inflammatory cell counts in the mucosal layer and corresponding counts in high-density mucosal regions. AI, artificial intelligence; CCC, Lin’s concordance correlation coefficient; ML, mucosal layer; SL, submucosal layer. Panels **(A–G)** show the agreement between AI-derived metrics (y-axis) and manual ground truth (x-axis) for lesion burden, inflammatory cell counts, and mucosal stromal proportion. Panel **(H)** compares two AI-based quantification approaches for mucosal inflammatory cell counts, with scatter plots, contour density estimates, and marginal distributions shown for plasma cells and lymphocytes. Higher CCC values and tighter clustering around the diagonal indicate better agreement.

The concordance between AI-generated quantitative measurements and pathologist evaluations is illustrated in **Figure 5**. Concordance correlation coefficients (CCC) demonstrated adequate agreement for crypt-level features: CCC was 0.783 for the crypt abscess ratio and 0.822 for the cryptitis ratio (**Figures 5A, B**). The concordance correlation coefficients (CCCs) for mucosal inflammatory cell metrics were 0.460 for plasma cell counts, 0.713 for lymphocyte counts, and 0.642 for the mucosal stromal ratio (**Figures 5C, D, G**).

Concordance was lower for some submucosal cell metrics. The CCC for plasma cell density in the submucosa was 0.376, whereas submucosal lymphocyte density showed moderate agreement (CCC 0.655; **Figures 5E, F**). Pairwise correlations between “whole-compartment” and “hotspot-based” cell density measures were generally moderate to strong (**Figure 5H**), indicating that hotspot metrics captured additional information while remaining consistent with global densities.

Because the CCC for submucosal plasma cell density was relatively low, we performed an exploratory bias analysis using the same evaluable paired cases included in the concordance analysis. AI-derived submucosal plasma cell density generally increased across higher manual ordinal grades, suggesting that the model retained sensitivity to greater plasma cell burden. However, AI-derived values overlapped substantially between manually negative and low-burden cases, indicating limited calibration in the lower-grade range. A standardized Bland-Altman analysis further showed wide limits of agreement between AI-derived continuous density and manual ordinal grading. Together, these findings suggest that the low CCC was mainly related to case-level variability and limited low-grade calibration, rather than consistent AI underestimation of high-burden plasma cell infiltration (**Supplementary Figure 1**).

### 3.4 Clinical correlations and comparison with non-Crohn’s colitis

Associations between AI-derived metrics and clinical indicators are presented in **Table 3**. In general, the observed correlations were typically weak, but several AI-derived histologic metrics were significantly associated with clinical variables. The crypt abscess ratio exhibited a mild positive correlation with endoscopic disease activity as measured by SES-CD (r = 0.255, p = 0.0001), whereas the cryptitis ratio exhibited an even weaker positive correlation with SES-CD (r = 0.133, p = 0.0488). CDAI demonstrated a statistically significant positive correlation with submucosal eosinophil density, the sole metric among the submucosal inflammatory cell measurements. However, the strength of this association was modest (r = 0.274, p = 0.0103). Conversely, the mucosa-to-submucosa plasma cell ratio was negatively correlated with CDAI (r = -0.303, p = 0.0079). Although this association was of limited magnitude, the mucosal stromal proportion also exhibited an inverse correlation with CRP (r = -0.200, p = 0.0003).

**Table 3:** Correlation analysis between AI-quantified metrics and clinical data.

Algorithm metric	CDAI		Activity		CRP	
	Correlation	P-value	Correlation	P-value	Correlation	P-value
Crypt Abscess	0.037	0.5097	0.255***	0.0001	-0.010	0.8516
Cryptitis	-0.089	0.1118	0.133*	0.0488	-0.064	0.2505
ML Stroma	-0.035	0.5292	0.002	0.9725	-0.200***	0.0003
ML Plasma Cells	-0.030	0.5912	0.091	0.1784	-0.104	0.0621
ML Lymphocytes	-0.103	0.0648	-0.103	0.1276	-0.079	0.1579
ML Eosinophils	-0.094	0.0909	0.042	0.5382	-0.052	0.3554
ML Neutrophils	-0.145**	0.0090	-0.062	0.3619	-0.093	0.0962
SL Plasma Cells	0.201	0.0623	0.051	0.6777	0.027	0.8071
SL Lymphocytes	0.012	0.9127	-0.153	0.2143	-0.198	0.0655
SL Eosinophils	0.274*	0.0103	0.040	0.7472	0.043	0.6903
SL Neutrophils	0.099	0.3633	0.113	0.3596	0.128	0.2378
Lymphocytes Ratio	-0.191	0.0990	-0.010	0.9393	-0.023	0.8457
Plasma Cells Ratio	-0.303**	0.0079	-0.135	0.3108	-0.169	0.1436

ML, mucosal layer; SL, submucosal layer; CDAI, Simplified Crohn’s Disease Activity Index; SES-CD, Simple Endoscopic Score for Crohn’s Disease; CRP, C-reactive protein.

Correlation coefficients and P values are shown for the associations between algorithm-derived histological metrics and the Simplified Crohn’s Disease Activity Index (CDAI), endoscopic activity, and C-reactive protein (CRP). “Activity” denotes endoscopic activity assessed by the Simple Endoscopic Score for Crohn’s Disease (SES-CD). Lymphocytes ratio and plasma cells ratio represent the ratio of cell counts in the mucosal layer to those in the submucosal layer.

Significance codes: *P < 0.05; **P < 0.01; ***P < 0.00.1.

In summary, these findings suggest that AI-derived histologic metrics are indicative of specific tissue-level characteristics that are linked to endoscopic and clinical disease activity. However, these quantitative outputs should be interpreted as complementary histologic measurements rather than isolated substitutes for CDAI, SES-CD, or systemic inflammatory biomarkers, given the low-to-moderate correlation coefficients.

**Figure 6:**
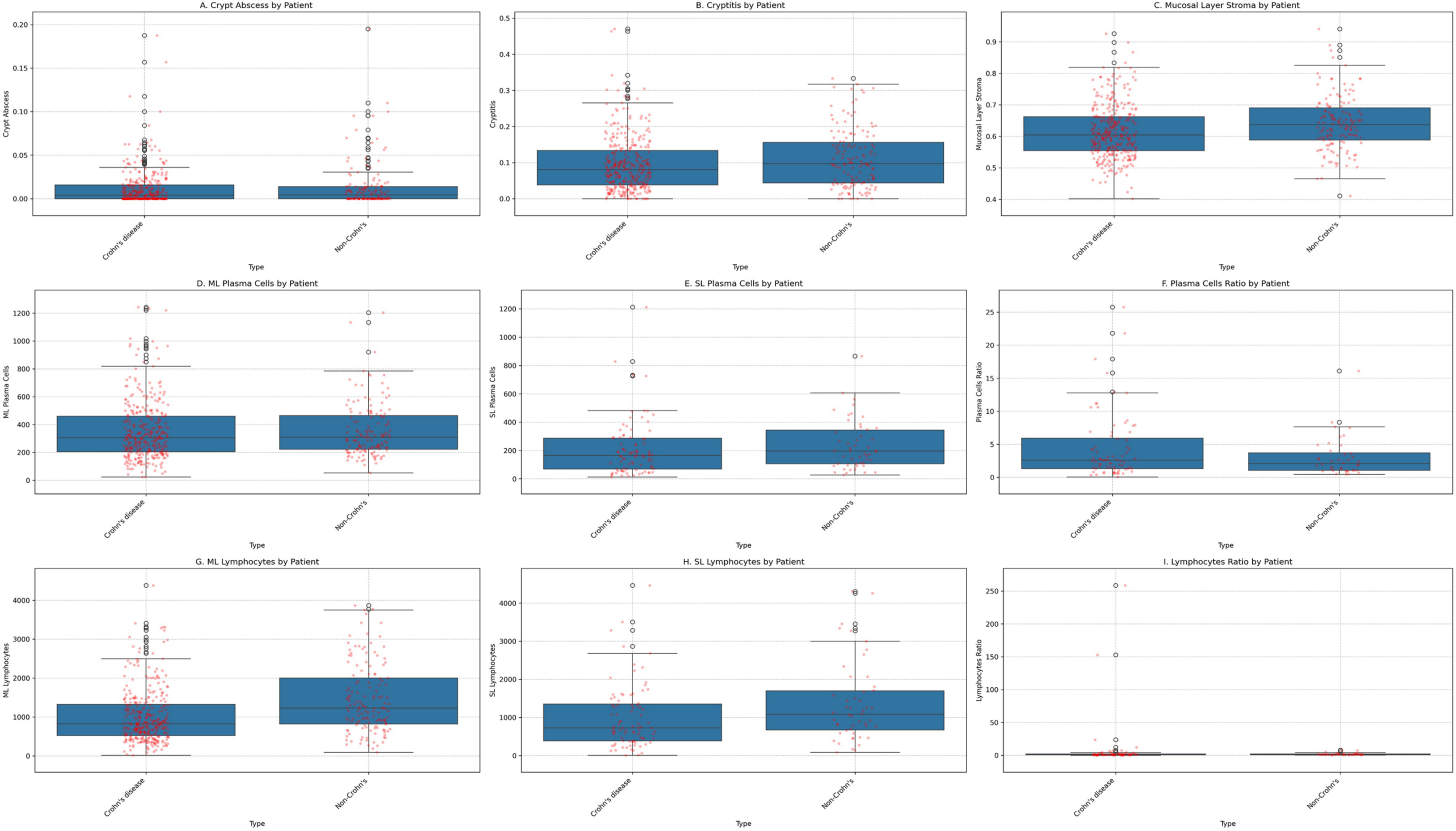
Comparison of AI-derived quantitative metrics in Crohn’s disease and non-Crohn’s lesions. **(A)** Box plot comparison of AI-derived crypt abscess metrics between Crohn’s disease (CD) and non-CD lesions. **(B)** Box plot comparison of AI-derived cryptitis metrics between CD and non-CD lesions. **(C)** Box plot comparison of mucosal stromal proportion between CD and non-CD lesions. **(D)** Box plot comparison of AI-derived plasma cell counts in the mucosal layer between CD and non-CD lesions. **(E)** Box plot comparison of plasma cell counts in the submucosal layer between CD and non-CD lesions. **(F)** Box plot comparison of the ratio of plasma cell counts between the mucosal and submucosal layers in CD versus non-CD lesions. **(G)** Box plot comparison of lymphocyte counts in the mucosal layer between CD and non-CD lesions. **(H)** Box plot comparison of lymphocyte counts in the submucosal layer between CD and non-CD lesions. **(I)** Box plot comparison of the ratio of lymphocyte counts between the mucosal and submucosal layers in CD versus non-CD lesions. AI, artificial intelligence; CD, Crohn’s disease; ML, mucosal layer; SL, submucosal layer. Each panel shows patient-level distributions of AI-quantified histological metrics, with box plots summarizing medians and interquartile ranges and overlaid scatter points representing individual patients. ML and SL plasma cell and lymphocyte counts are expressed as the number of cells per unit mucosal or submucosal area, respectively. Ratio metrics (panels **(F, I)**) are defined as the ML-to-SL cell count ratio for plasma cells and lymphocytes. Higher values indicate greater inflammatory burden or stromal involvement within the corresponding compartment.

**Figure 6** compares AI-derived metrics between Crohn’s disease and non-Crohn’s inflammatory colitis. Cryptitis and crypt abscess burdens were elevated in both groups and did not clearly discriminate CD from non-Crohn’s colitis (**Figures 6A, B**). Non-Crohn’s cases had higher mucosal stromal ratios than CD cases (**Figure 6C**). By contrast, CD biopsies showed more pronounced submucosal inflammatory cell infiltration, with higher submucosal plasma cell and lymphocyte densities and a higher submucosa-to-mucosa inflammatory cell density ratio (**Figures 6D–I**). In this exploratory comparison, CD biopsies showed relatively greater submucosal inflammatory cell infiltration than the heterogeneous non-CD inflammatory colitis group. Because the comparator group included several inflammatory colitis entities (**Supplementary Table 1**), these findings should be interpreted as exploratory observations of compartment-specific inflammatory patterns rather than as evidence supporting a diagnostic classifier.

## 4 Discussion

### 4.1 Principal findings

In this study we established a hierarchical, AI-driven framework for multi-scale quantitative analysis of endoscopic biopsy slides from patients with Crohn’s disease. By automating segmentation of mucosal and submucosal compartments, delineation and classification of individual crypts, and detection of specific inflammatory cell populations, the pipeline generated a set of interpretable quantitative metrics that are difficult to obtain reproducibly with conventional histopathology.

The models showed high technical performance, with Dice scores tightly clustered near 1.0 for crypts and major tissue compartments and AUCs >0.95 for key classification tasks such as cryptitis and crypt abscesses. AI-derived cryptitis and crypt abscess ratios demonstrated good concordance with pathologist grading (CCC = 0.78-0.82), while concordance for several cell-level metrics was moderate, particularly for plasma cell counts in the mucosa and submucosa. Importantly, these metrics were not only technically robust but also clinically informative: submucosal inflammatory cell density correlated positively with CDAI, the mucosa-to-submucosa inflammatory cell density ratio correlated inversely with CDAI, mucosal stromal proportion correlated inversely with CRP, and submucosal-predominant inflammation distinguished Crohn’s disease from non-Crohn’s colitis in this cohort.

### 4.2 From subjective grading to objective quantification

This work’s primary contribution is the conversion of previously subjective histological perceptions into objective, reproducible quantitative data. Histologic evaluation of Crohn’s disease activity and chronicity generally depends on semi-quantitative grading and subjective impressions, which are recognized to exhibit inter-observer variability, particularly concerning subtle characteristics like basal plasmacytosis, focal cryptitis, and low-grade chronic inflammation (3, 10). Our findings indicate that a deep learning framework can consistently identify these essential components-mucosal architecture, crypt lesions, and inflammatory infiltrates-with high accuracy, establishing a robust basis for subsequent quantification (12).

The strong correlation between AI-derived cryptitis and crypt abscess ratios and pathologist grading indicates that automated measurement of these recognized markers of active inflammation is viable and may mitigate heterogeneity in standard reporting (18, 30). The moderate agreement shown for certain cell-level metrics, especially plasma cell densities, presumably indicates not a deficiency in the model but rather the inherent challenges encountered by human observers in comprehensively quantifying dispersed cell populations over extensive tissue regions. In routine practice, pathologists frequently depend on hotspot-based estimates; in contrast, our AI conducts systematic, cell-by-cell enumeration over the whole segmented compartment. The moderate-to-strong associations identified between whole-compartment and hotspot-based AI densities reinforce this interpretation and imply that whole-slide AI quantification may ultimately yield a more thorough assessment of inflammatory load than human visual sampling.

### 4.3 Submucosal-predominant inflammation as a quantitative CD signature

Perhaps the most clinically meaningful observation from this study is the central role of submucosal inflammation. Because endoscopic biopsies frequently sample only superficial tissue, the submucosa has traditionally been under-evaluated, despite the well-recognized transmural nature of Crohn’s disease. Our hierarchical approach, which explicitly segments the submucosal compartment, allowed us to derive submucosal inflammatory cell density and a submucosa-to-mucosa inflammatory cell ratio.

The positive correlation between submucosal inflammatory cell density and CDAI, together with the inverse relationship between the mucosa-to-submucosa density ratio and CDAI, indicates that deeper inflammatory burden is more closely linked to clinical disease activity than mucosal inflammation alone. This is consistent with the concept that Crohn’s disease is fundamentally a transmural process and suggests that even relatively small amounts of submucosa in endoscopic biopsies can carry important information about systemic disease activity (31).

The comparison with non-Crohn’s inflammatory colitis further underscores the diagnostic relevance of this “depth-of-inflammation” gradient. While cryptitis and crypt abscesses were common to both CD and non-CD colitis and therefore non-discriminatory, Crohn’s disease was characterized by higher submucosal plasma cell and lymphocyte densities and a higher submucosa-to-mucosa inflammatory cell ratio. These findings quantitatively support the long-held notion that disproportionate involvement of deeper layers is characteristic of CD (32) and suggest that AI-derived submucosal metrics may serve as useful adjunctive biomarkers in challenging differential diagnoses within the IBD spectrum.

It’s also intriguing that there is an inverse relationship between the fraction of mucosal stromal and CRP. While causality cannot be established, an elevated stromal ratio may indicate more chronic or fibrotic remodeling accompanied by less cellular inflammation, consistent with lower systemic inflammatory markers (33). This metric may consequently augment activity-focused metrics by elucidating structural damage and chronicity, pertinent for surgical planning and long-term prognosis.

### 4.4 Clinical implications

From a clinical perspective, objective histologic metrics derived from routine biopsies could complement endoscopic indices by providing standardized tissue-level information on inflammatory burden and structural remodeling (2, 34). In particular, compartment-specific inflammatory densities and mucosa-to-submucosa ratios may further clarify the depth and distribution of inflammation, although these metrics should be interpreted together with clinical, biochemical, and endoscopic findings rather than used as standalone indicators (31, 35).

In practical diagnostic workflows, the proposed AI system is intended to function as a pathologist-supervised decision-support tool rather than an autonomous diagnostic system. At the diagnostic workstation, the platform could generate two complementary outputs. First, it provides structured quantitative tables summarizing cryptitis ratio, crypt abscess ratio, mucosal and submucosal inflammatory cell densities, hotspot inflammatory cell densities, and mucosal stromal proportions. These values could be incorporated into structured pathology reports as objective adjunctive measurements. Second, the platform generates spatial heatmaps that highlight inflammatory hotspots, allowing pathologists to verify algorithmic outputs, prioritize regions requiring focused review, and interpret quantitative findings in their histologic context. This visual review step is particularly important for metrics with limited calibration, such as submucosal plasma cell density in low-burden regions. Such tools may be especially valuable in centers with limited IBD subspecialty expertise or high workloads (36–38). Over time, embedding quantitative metrics in routine histology reports could facilitate multicenter trials, where harmonized histologic endpoints are essential, and enable more consistent communication between pathologists, gastroenterologists, and surgeons.

### 4.5 Strengths and limitations

Strengths of this study include a relatively large, well-characterized cohort of patients with Crohn’s disease, systematic digitization of endoscopic biopsies, and a biologically informed, multi-scale AI design that mirrors routine histopathologic reasoning while integrating technical validation, expert comparison and linkage to clinical indices and non-Crohn’s inflammatory colitis (39).

Several limitations should nevertheless be acknowledged. First, the study was a single-center retrospective study, and model performance may vary across institutions because of differences in patient populations, tissue processing, H&E staining, and scanner platforms. Although slide-level quality control and color augmentation were applied, prospective multicenter external validation remains necessary before broader clinical implementation. Second, ground-truth labels were based on expert human annotation, which is inherently imperfect, particularly for submucosal inflammatory cell quantification. This was most evident for submucosal plasma cell density, which showed limited agreement with manual assessment. The supplementary grade-stratified calibration analysis suggested relative AI over-detection in manually negative or low-burden submucosal regions and limited calibration between adjacent low-grade categories. Therefore, this metric should be interpreted cautiously, especially in low-burden cases, and should be reviewed together with heatmap visualization under pathologist supervision rather than used as a standalone endpoint.

Third, although several AI-derived histologic metrics were statistically associated with CDAI, SES-CD, or CRP, the correlation coefficients were generally weak to modest. These metrics, therefore, should not be interpreted as direct surrogates for clinical activity, endoscopic severity, or systemic inflammation, but rather as complementary tissue-level quantitative descriptors within a broader clinicopathologic framework. Fourth, the non-Crohn’s inflammatory colitis group was diagnostically heterogeneous. Although its composition was summarized in the **Supplementary Materials**, the comparison with Crohn’s disease should be regarded as exploratory rather than diagnostic. Larger and more balanced disease-specific comparator cohorts are needed to determine whether compartment-specific AI-derived inflammatory patterns can assist differential diagnosis.

Finally, the current framework focused on cryptitis, crypt abscesses, inflammatory cell densities, and mucosal stromal proportion but did not model other important Crohn’s disease-related features, such as granulomas, fissuring ulcers, transmural inflammation, neural hyperplasia, and fibrosis (40). Future studies should pursue prospective multicenter validation, longitudinal assessment of AI-derived metrics in relation to treatment response and outcomes, expansion to additional Crohn’s disease-specific histologic features, and integration with endoscopic, radiologic, molecular, and clinical data (41, 42).

## 5 Conclusion

In summary, we developed an interpretable deep learning framework that enables objective, reproducible quantification of key histopathologic features in Crohn’s disease endoscopic biopsies and yields metrics that track clinical disease activity while capturing submucosal-predominant inflammation as a distinguishing pattern. Prospective multicenter and longitudinal validation will be essential to confirm its clinical utility and support its integration into precision, data-driven management of inflammatory bowel disease.

## Data Availability

The raw data supporting the conclusions of this article will be made available by the authors, without undue reservation.
