# Supplementary material for: Deep learning-based quantitative histopathology of endoscopic biopsies in Crohn’s disease: a retrospective cross-sectional validation study
Source: Front Immunol. 2026 Jun 5;17:1841261. doi: 10.3389/fimmu.2026.1841261 (PMC13279501; doi:10.3389/fimmu.2026.1841261)
Supplement: Supplementary file 1 [file Supplementaryfile1.docx]

**Supplementary Table S1.** **Diagnostic composition of the exploratory non-CD inflammatory colitis comparator group**

| Diagnostic category | Patients, n (%) | Slides, n(%) | Predominant biopsy site(s) |
| --- | --- | --- | --- |
| Ulcerative colitis | 316 (49.1) | 1568 (52.6) | Colon/rectum |
| Infectious colitis | 56 (8.7) | 280 (9.4) | Colon |
| Ischemic colitis | 75 (11.7) | 392 (13.1) | Colon |
| Drug-induced colitis | 22 (3.4) | 132 (4.4) | Colon |
| Nonspecific chronic colitis | 140 (21.8) | 461 (15.5) | Ileocolonic |
| Other inflammatory colitis | 34 (5.3) | 150 (5.0) | Colon |
| Total | 643 (100.0) | 2,983(100.0) | - |

Notes. Diagnostic categories were assigned according to the final clinicopathologic diagnosis based on clinical, endoscopic, radiologic, and histopathologic information. The non-CD inflammatory colitis group was not used for model development, training, or internal validation, and was included only for exploratory comparison of AI-derived compartment-specific inflammatory patterns. This comparator analysis was not intended to establish or validate a diagnostic classifier. Biopsy site refers to the predominant sampled site recorded in the pathology archive. CD, Crohn’s disease.
