# Supplementary material for: Deep learning-based quantitative histopathology of endoscopic biopsies in Crohn’s disease: a retrospective cross-sectional validation study
Source: Front Immunol. 2026 Jun 5;17:1841261. doi: 10.3389/fimmu.2026.1841261 (PMC13279501; doi:10.3389/fimmu.2026.1841261)
Supplement: Supplementary file 2 [file Supplementaryfile2.docx]

**Supplementary figure S1. Exploratory Bias Analysis of AI-Derived Submucosal Plasma Cell Assessment**


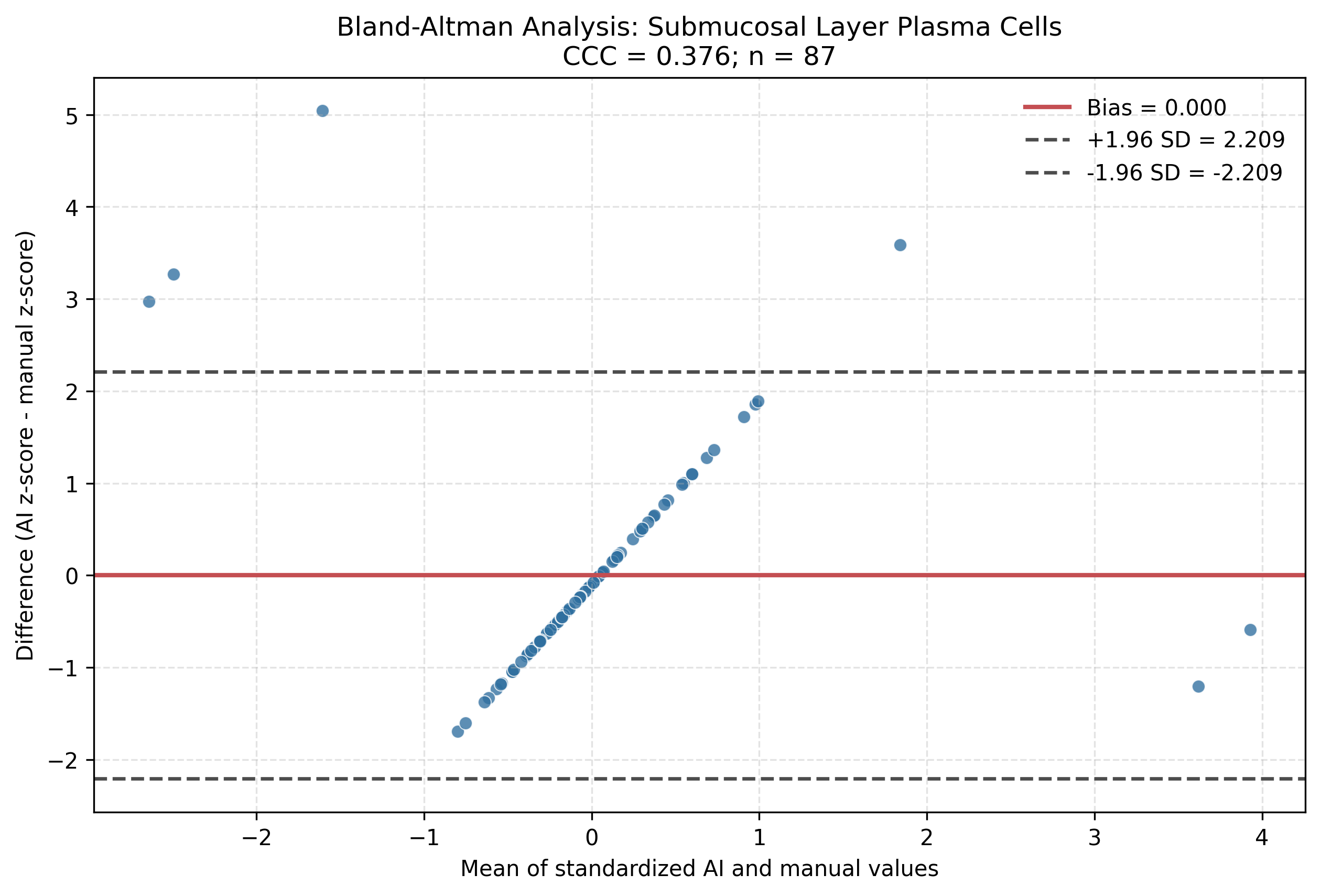


Notes. This exploratory standardized Bland–Altman analysis compared AI-derived submucosal plasma cell density with manual ordinal pathological grading. Because manual assessment was recorded as an ordinal grade rather than an absolute cell-density measurement, both AI-derived density and manual grade were standardized before paired differences were calculated. The red solid line indicates the mean standardized difference, and the dashed lines indicate the 95% limits of agreement. The analysis used the same evaluable paired cases included in the concordance analysis for this endpoint, restricted to slides with identifiable submucosa and non-missing AI/manual assessments. The mean standardized difference was 0.000, and the 95% limits of agreement ranged from −2.209 to 2.209. Because the paired values were standardized, the mean difference should not be interpreted as evidence of the absence of absolute measurement bias. The wide limits of agreement indicate case-level variability between AI-derived continuous density and manual ordinal grading, supporting cautious interpretation of this metric as a complementary quantitative output.
